# Prevalence of impaired flow-mediated epicardial vasodilation among different types of coronary flow regulation

**DOI:** 10.1007/s10554-026-03736-3

**Published:** 2026-06-06

**Authors:** Ines Valenta, Mohammad Alavi, Ashwin S. Parihar, Sudhir Jain, Thomas Hellmut Schindler

**Affiliations:** 1 Mallinckrodt Institute of Radiology, Division of Nuclear Medicine - Cardiovascular, Washington University in St. Louis School of Medicine, St. Louis, MO USA; 2 Department of Internal Medicine, Cardiovascular Division, Washington University in St. Louis School of Medicine, John T. Milliken, St. Louis, MO USA; 3 Mallinckrodt Institute of Radiology - Division of Nuclear Medicine, Washington University in St. Louis, Cardiovascular. 510 S. Kingshighway Boulevard, Campus Box 8223, St. Louis, MO 63110 USA

**Keywords:** Cardio-metabolic risk, Coronary microvascular function, Endothelium, Epicardial vasomotion, Flow gradient, Myocardial blood flow, Myocardial flow reserve, Myocardial perfusion, Positron emission tomography

## Abstract

To assess the prevalence of impairment of flow-mediated epicardial vasodilation (IEV) in the presence of normal and abnormal coronary microvascular function. In 332 consecutive symptomatic patients, global and longitudinal myocardial blood flow (MBF) at rest and during pharmacologically stimulated hyperemia was evaluated with ^13^N-ammonia positron emission tomography/computed tomography (PET/CT). Normal coronary microvascular function (nCMF) was defined by a myocardial flow reserve (MFR = MBFstress/MBFrest) of ≥ 2.0 (group 1; *n* = 210 (G1)), while an abnormal MFR of < 2.0 (predominantly due to decreases in hyperemic MBF) denoted classical CMD (group 2; *n* = 83 (G2)) or (predominantly related to increases in resting MBF ≥ 1.0mL/g/min) signified endogen CMD (group 3; *n* = 39 (G3)), respectively. Furthermore, normal flow-mediated epicardial vasodilation (NEV) was defined as longitudinal hyperemic MBF gradient < -0.10 mL/g/min, whereas a value ≥ -0.10 mL/g/min signified IEV.In the entire study population, IEV was present in 34.6.% (115/332). IEV was highest in group 1 (46.2%), while there was a comparable low prevalence between group 2 and group 3 (13.3% vs. 17.9%; *p* = 559). The hyperemic longitudinal MBF gradient was significantly higher in G1 compared to G2 (-0.28 ± 0.10 vs. -0.19 ± 0.08 mL/g/min; *p* ≤ 0.008), but comparable between group 1 and group 3 (-0.28 ± 0.10 and − 0.27 ± 0.10 mL/g/min; *p* = 0.795). The hyperemic longitudinal MBF gradient and ΔMBF Gradient correlated inversely with the global hyperemic MBF, respectively (*r* = 0.41, SEE = 0.09 and *r* = 0.34, SEE = 0.09, both *p* ≤ 0.001). Nearly half of symptomatic patients with normal global MFR may have IEV. As IEV carries important diagnostic and prognostic information, it may further optimize prognostication on cardiovascular individuals deserving further clinical testing.

**Graphical abstract:**

**Figure:**
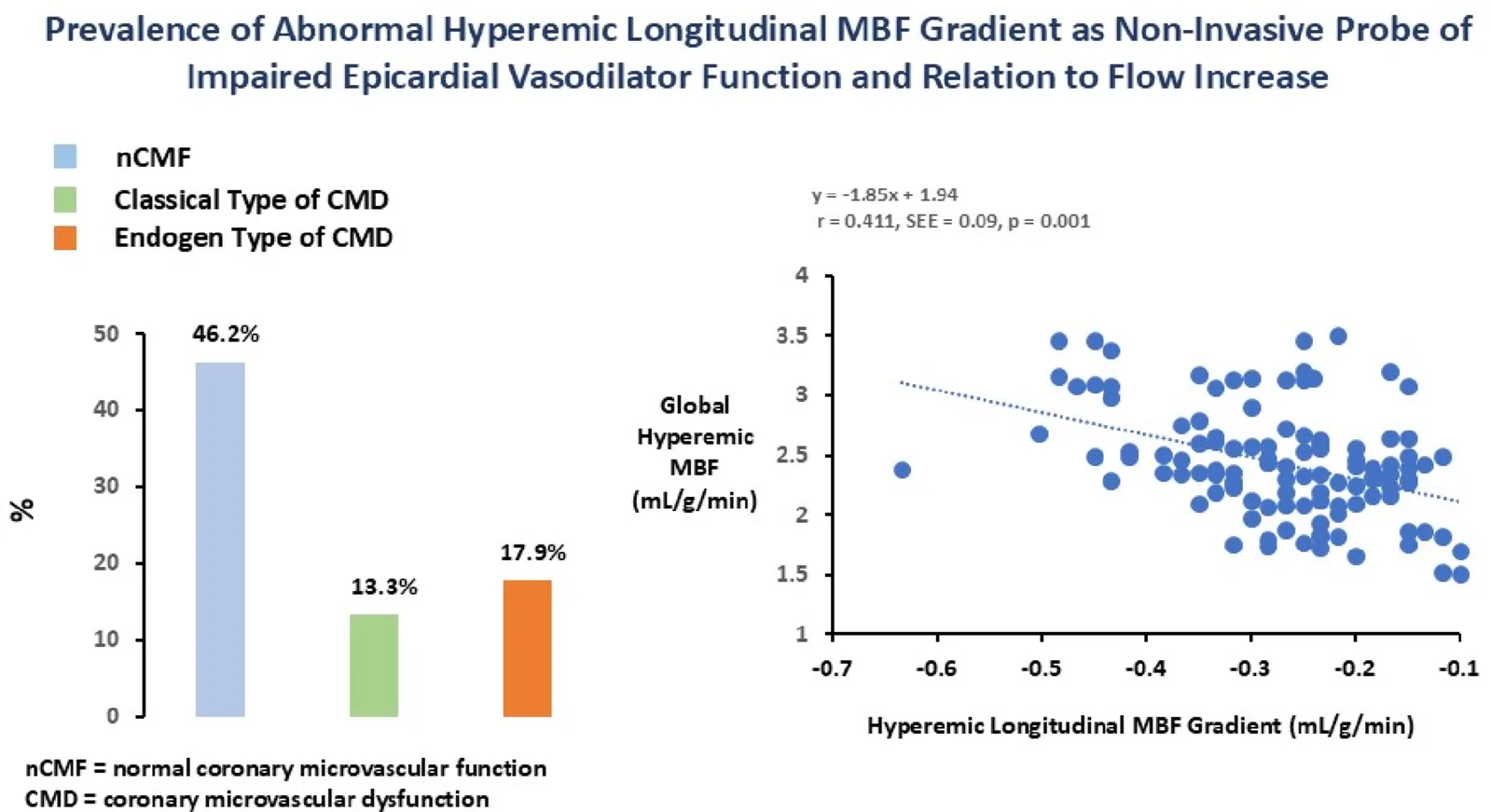

## Introduction

Normal flow-mediated and thus endothelium-dependent coronary vasodilation confers pivotal anti-atherosclerotic and anti-thrombotic effects [1, 2]. Numerous invasive investigations in the assessment of coronary vasomotion [3, 4] have unraveled an impairment of endothelium-dependent coronary function as an independent predictor not only of the initiation and/or advancement of CAD but also worse cardiovascular outcome. Until now, the specific assessment of endothelium-dependent epicardial function needed to be performed invasively during coronary angiography needing specifically acetylcholine-stimulated endothelial release of nitric oxide or flow-mediated and thus endothelium-dependent vasodilation during hyperemic coronary flow via intracoronary application of adenosine, or via sympathetic stress stimulation with cold pressor testing [5–7]. Conversely, the invasive nature of coronary angiography hampers a widespread clinical application of the assessment of the regulation of coronary vasomotor tone [2, 8]. Clinical PET flow studies [1, 9–13] have demonstrated that the non-invasive identification of reductions in myocardial flow reserve or coronary microvascular dysfunction (CMD) may helpful to predict cardiovascular outcome in patients with and without obstructive CAD. In recent years, a new concept of PET-determined abnormal decreases of MBF from the base to apical direction of the left ventricle or so called longitudinal MBF gradient during hyperemic flow stimulation has been put forth to reflect down-stream, hemodynamic effects of diffuse CAD and/or impaired flow-mediated epicardial vasodilation in cardiovascular risk individuals [2, 14–16]. Such a non-invasive approach may prove useful to identify early functional and/or structural alterations of the epicardial artery, where the CAD process takes place, that otherwise may be missed by the assessment of global hyperemic MBF and/or myocardial flows reserve (MFR) alone [2, 16, 17]. The prevalence of impaired flow-mediated epicardial vasodilation (IEV) in the presence of normal coronary microvascular function (nCMF) and different types of CMD remains to be further elucidated.

In this respect, we strived to assess the prevalence of impairment of flow-mediated epicardial vasodilation (IEV) in the presence of nCMF, classical and endogen type of CMD and its relation to hyperemic flow increases in a cardiometabolic risk population.

## Materials and methods

### Study population

The study population comprised 332 symptomatic patients (132 men and 200 women, mean age, 60 ± 13years) with predominant metabolically unhealthy obesity having a low likelihood of flow-limiting obstructive CAD and widely medically controlled cardiovascular risk factors such as dyslipidemia, arterial hypertension, and/or type 2 diabetes mellitus (Table 1). These patients were consecutively referred for rest and pharmacologically induced stress myocardial perfusion and myocardial blood flow (MBF) assessment with ^13^N-ammonia positron emission tomography/computed tomography (PET/CT) to evaluate the presence of obstructive CAD and/or CMD between May 2023 and December 2025. Patients were only included for study analysis, if they had normal rest and pharmacologic stress ^13^N-ammonia PET/CT myocardial perfusion images as well as normal wall motion analysis on gated ^13^N-ammonia PET that widely excluded significant obstructive CAD [2, 15]. Consequently, patients with hemodynamically significant obstructive CAD lesions but also those patients with peak stress transient ischemic cavity dilation of the left ventricle during maximal vasomotor stress on gated PET images, as a reflection of stress-induced diffuse myocardial ischemia owing to hemodynamically obstructive left main and/or multivessel CAD, as well as congestive heart failure patients were not included in this final analysis.

**Table 1:** Patient characteristics among groups 1–3

*N* (numbers)	Group 1	Group 2	*p* Value	Group 3	*p* Value
	210	83		39	
Sex (female/male)	142/68	29/54	0.0001	29/10	0.304
BMI, kg/m^2^	36.3 ± 9.98	35.7 ± 9.12	0.317	37.9 ± 10.1	0.344
Age, yrs	58.4 ± 12.4	66.9 ± 12.4	0.001	60.8 ± 13.3	0.307
CCS	0.95 ± 1.54	2.74 ± 2.03	0.001	1.38 ± 1.64	0.139
Cardiovascular risk factors					
Hypertension, n	156 (74.3)	74 (89.2)	0.340	38 (97.4)	0.279
Dyslipidemia, n	144 (68.5)	73 (87.9)	0.197	26 (66.6)	0.918
Diabetes mellitus, n	60 (28.6)	48 (57.8)	0.002	16 (41.0)	0.272
Smoking, n	15 (7.2)	9 (10.8)	0.341	3 (7.6)	0.910
Family history of CAD, n	18 (8.6)	8 (9.6)	0.792	2 (5.1)	0.497
Obesity, n	143 (68.1)	60 (72.2)	0.766	34 (87.2)	0.338
Morbid obesity, n	78 (37.1)	33 (39.7)	0.780	16 (41.0)	0.759
Lipid status					
Total cholesterol, mg/dl	159 ± 36	143 ± 44	0.008	155 ± 38	0.479
LDL cholesterol, mg/dl	84 ± 34	77 ± 39	0.138	80 ± 33	0.439
HDL cholesterol, mg/dl	54 ± 17	44 ± 16	0.001	56 ± 19	0.871
Triglyceride, mg/dl	125 ± 70	131 ± 63	0.603	132 ± 88	0.685
Glucose, mg/dl	116 ± 37	123 ± 46	0.161	125 ± 46	0.247
hs CRP, mg/l	9.41 ± 15.61	21.05 ± 32.45	0.006	13.06 ± 18.48	0.407
HbA_1c_, %	6.02 ± 1.43	6.65 ± 1.36	0.003	6.22 ± 1.24	0.436

Values are mean ± standard deviation (SD). Numbers (%). P values versus group 1 (G1) (Mann-Whitney U test for independent samples)

BMI = body mass index; CCS = coronary artery calcium score; LDL = low-density lipoprotein; HDL = high-density lipoprotein;

hsCRP = high-sensitivity C-reactive protein; HbA1c = glycosylated hemoglobin

**Fig. 1:**
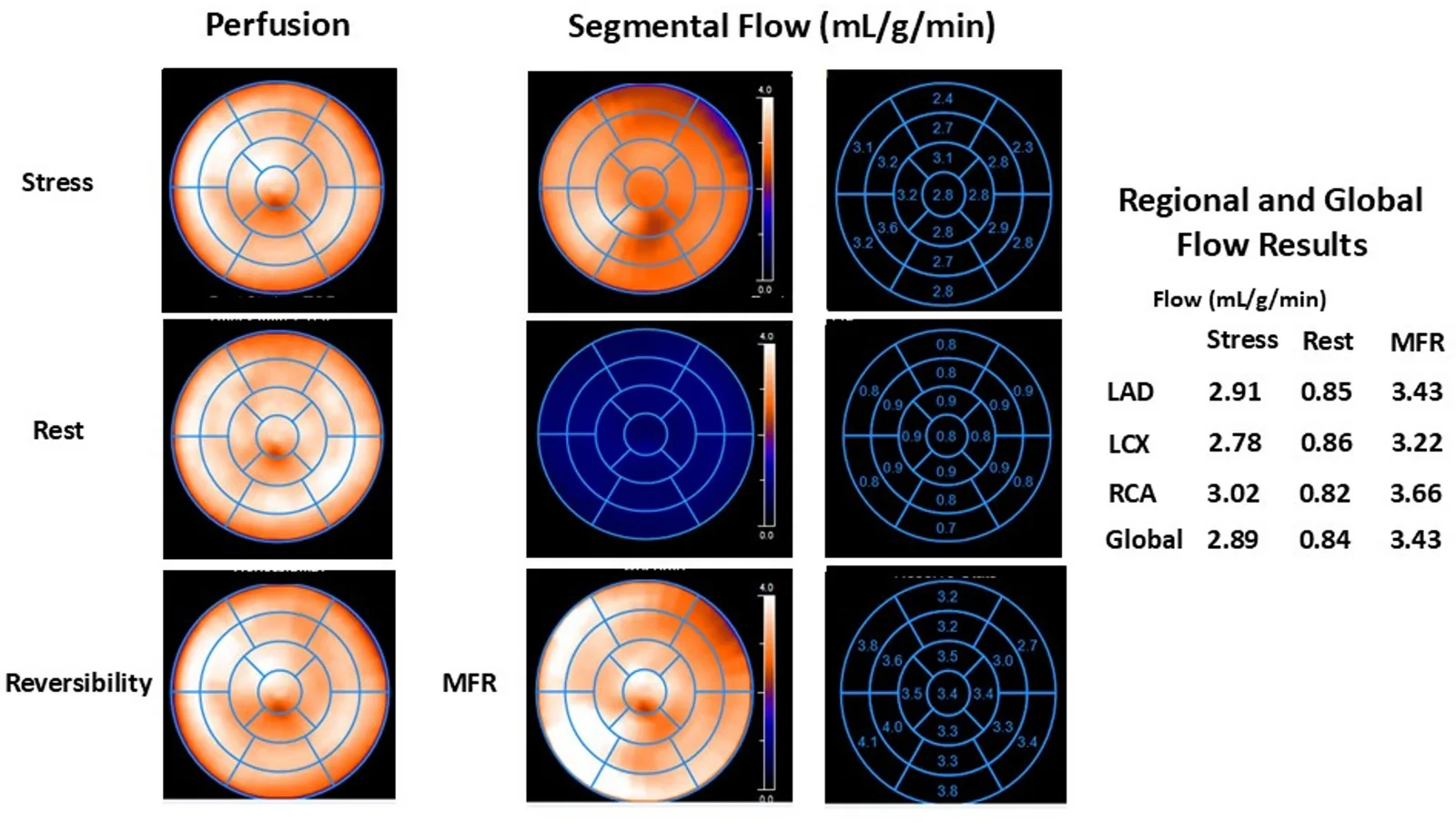
Normal Myocardial Perfusion, Global Myocardial Flow Reserve (MFR), and Myocardial Blood Flow (MBF) Study with ^13^N-ammonia PET/CT. This 44-year-old woman presented with atypical chest discomfort, and dyslipidemia. There is normal and homogenous ^13^N-ammonia uptake of the left ventricle at rest and regadenoson-stress PET/CT count images with corresponding normal myocardial perfusion on polar map display widely ruling out hemodynamically significant obstructive CAD *(right panel)*. Regional MBF quantification denotes normal hyperemic MBFs and MFR in all three major coronary artery territories of the LAD, LCx, and RCA with a global hyperemic MBF of 2.89 mL/g/min, global rest MBF of 0.84 mL/g/min, and corresponding global MFR of 3.43 signifying normal coronary microvascular function (nCMF) *(middle panel)*. Subsequently, longitudinal MBFs in the mid to mid-to-distal myocardial segments of the LV corresponding to the vascular territories of the LAD (segments:7–8 and 13–14), Lcx (segments:11–12 and 16), and RCA (segments:9–10 and 15) were determined applying a 17-segment model. In this case, LAD mid-segments 7 and 8 with stress MBF of 3.2 and 2.7 mL/g/min (mean:2.95 ml/g/min), but without a decline in the mid-to-distal segments 13–14 with stress MBF of 3.2 and 3.1 mL/g/min (mean: 3.05 mL/g/min) denoting the absence of a hyperemic abnormal longitudinal MBF gradient in the LAD distribution. This can also be widely appreciated in the LCx distribution, mid-segments (11 and 12) with stress MBF of 2.8 and 2.9 mL/g/min) (mean: 2.85 mL/g/min), and mid-to-distal segment (16) with stress MBF of 2.8 mL/g/min. Conversely, for the RCA distribution, mid-segments (9 and 10) demonstrate stress MBF of 2.7 and 3.6 mL/g/min (mean: 3.15 mL/g/min) and mid-to-distal segment (15) with stress MBF of 2.8 mL/g/min, respectively, resulting in a regional MBF difference or abnormal longitudinal MBF gradient of −0.35 mL/g/min. When computing the mean stress MBF values for the mid and mid-to-distal segments of the LAD, LCx, and RCA distribution on a patient level, however, then the corresponding mean mid-segment stress MBF flow of 2.97 mL/g/min was virtually similar to the mean mid-to-distal stress MBF flow of 2.98 denoting the absence of an abnormal longitudinal MBF gradient during hyperemic MBF stimulation *(left panel)*

**Fig. 2:**
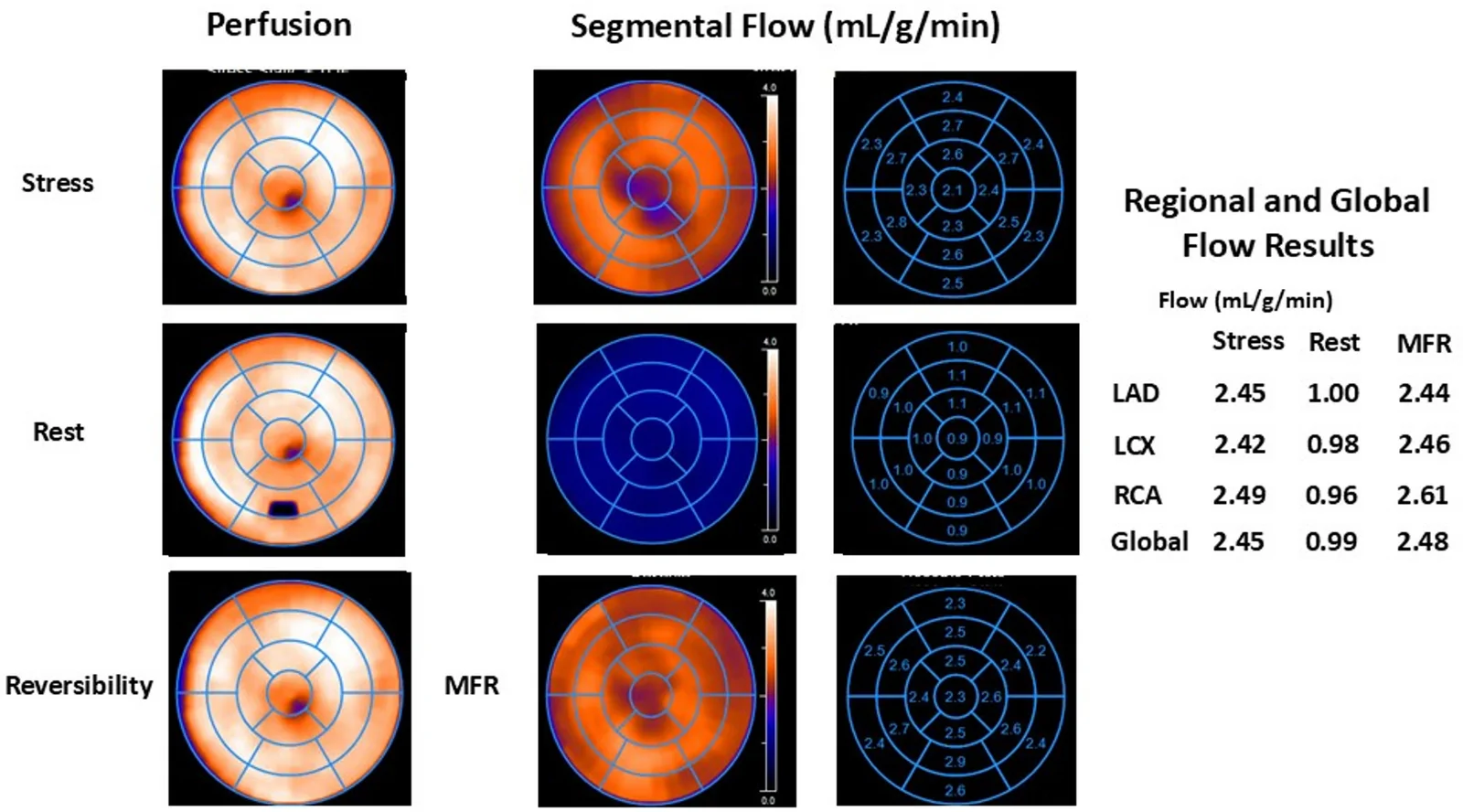
Normal Myocardial Perfusion, Global MFR but Abnormal Hyperemic Longitudinal MBF Gradient with ^13^N-ammonia PET/CT. This 68-year-old woman presented with atypical chest discomfort, dyslipidemia and arterial hypertension. As can be appreciated, there is widely normal and homogenous ^13^N-ammonia uptake of the left ventricle at rest and regadenoson-stress PET/CT count images with corresponding normal myocardial perfusion on polar map display widely excluding significant flow-limiting obstructive focal CAD *(right panel)*. Regional MBF quantification denotes normal hyperemic MBFs and MFR in all three major coronary artery territories of the LAD, LCx, and RCA with a global hyperemic MBF of 2.45 mL/g/min, global rest MBF of 0.99 mL/g/min, and corresponding global MFR of 2.48 signifying normal nCMF *(middle panel)*. Subsequently, longitudinal MBFs in the mid to mid-to-distal myocardial segments of the LV corresponding to the vascular territories of the LAD (segments:7–8 and 13–14), Lcx (segments:11–12 and 16), and RCA (segments:9–10 and 15) were determined applying a 17-segment model. In this patient, LAD mid-segments 7 and 8 with stress MBF of 2.7 and 2.7 mL/g/min (mean:2.7 ml/g/min) with a decrease in the mid-to-distal segments 13–14 with stress MBF of 2.3 and 2.6 mL/g/min (mean: 2.45 mL/g/min) signifying an abnormal hyperemic abnormal longitudinal MBF gradient of −0.25 mL/g/min in the LAD distribution. Similarly, in the LCx distribution, mid-segments (11 and 12) with stress MBF of 2.7 and 2.5 mL/g/min (mean: 2.60 mL/g/min), and mid-to-distal segment (16) with stress MBF of 2.4 mL/g/min, resulting in an hyperemic abnormal longitudinal MBF gradient of −0.20 mL/g/min. As regards the RCA distribution, mid-segments (9 and 10) demonstrate stress MBF of 2.6 and 2.8 mL/g/min (mean: 2.70 mL/g/min) and mid-to-distal segment (15) with stress MBF of 2.3 mL/g/min, respectively, denoting also a hyperemic abnormal longitudinal MBF gradient of −0.40 mL/g/min. When computing the mean stress MBF values for the mid and mid-to-distal segments of the LAD, LCx, and RCA distribution on a patient level, however, then the corresponding mid-segment stress MBF flow of 2.67 mL/g/min was markedly higher than the mean mid-to-distal stress MBF flow of 2.40 mL/g/min resulting in a mean abnormal hyperemic longitudinal MBF gradient of −0.27 mL/g/min *(left panel)*

^13^N-ammonia PET/CT concurrently assessed myocardial perfusion and calculated global and longitudinal myocardial blood flow (MBF) at rest and during pharmacologically stimulated hyperemia. Normal coronary microvascular function (nCMF) (group 1) was defined by a myocardial flow reserve (MFR = MBFstress/MBFrest) of ≥ 2.0 (Fig. 1), while an abnormal MFR of *<* 2.0 denoted CMD (Fig. 2) [2]. According to a JACC Cardiovascular Imaging expert panel statement [1], CMD was then subgrouped into “classical type” of CMD (predominantly related to decreases in hyperemic MBFs with < 2.0 mL/g/min) (group 2) and “endogen type” of CMD (predominantly related to increases in resting MBF and hyperemic MBF ≥ 2.0 mL/g/min) (group 3). In addition, segmental MBFs in the mid and mid-distal myocardial segment of the left ventricle corresponding to the vascular territories of the LAD, LCx and RCA were assessed [2]. A change in MBF from mid to mid-distal LV myocardium was defined as MBF difference to denote a longitudinal MBF gradient (ml/g/min). Changes in MBF difference from baseline to hyperemia were defined as ∆MBF difference (MBF difference during hyperemia minus MBF difference at rest) to signify ∆longitudinal MBF gradient [16, 18]. Vasoactive medications such as calcium channel blockers, angiotensin-converting enzyme inhibitors, angiotensin II receptor blockers, statin as well as b-blockers, and diuretics were withheld at least 24 h before PET/CT myocardial perfusion-flow examination [19, 20]. All study patients refrained from caffeine-containing beverages for at least 24 h and from smoking for at least 12 h prior to the cardiac PET/CT study.

### Ethical approval

The study was approved by the Washington University in St. Louis (No.201812037), and each participant signed a clinical approved informed consent form.

### Cardiac ^13^N-ammonia PET/CT study protocol

An initial topogram to define the axial field of view and a low-dose CT scan (120 kV, 30 mA) for attenuation correction we perfomed. Subsequently, ^13^N-ammonia PET determined myocardial perfusion and MBF in ml/min/g with serially acquired images (Biograph mCT PET-CT scanner, Siemens Healthineers, Erlangen, Germany) and a two-compartment tracer kinetic model at rest and during regadenoson-stimulated hyperemia was performed. PET ten-minutes list mode data acquisition during regadenoson-stimulated hyperemia (0.4 mg intravenous bolus injection over 10- and 20-seconds interval) was conducted immediately after injection of ≈ 259 MBq and ≈ 518 MBq ^13^N-ammonia at rest and during pharmacologic stress, respectively, as a bolus and subsequent infusion of 10 mL saline solution over 30 s (0.33 mL/sec) via infusion pump (e.g. Aitecs Syringe Pump) [20].

Myocardial perfusion at rest and during pharmacologic-induced hyperemia was evaluated visually on static reoriented short-and long-axis myocardial images and semi quantitatively on the corresponding polar map from the last static 10-min trans axial PET image. Semiquantitative evaluation of ^13^N-ammonia PET perfusion images was conducted with a standard 17-segment model and a five-point grading system by two expert observers. Summed stress score (SSS), summed rest score (SRS), and summed difference score (SDS) were obtained. Only patients with normal rest-stress myocardial perfusion as denoted by a SDS *<* 2, and normal wall motion on gated PET with a LVEF ≥ 45% were selected for study analysis. Image interpretation was visually conducted in consensus by two experienced nuclear cardiovascular imaging experts. In the case of disagreement among readers, consensus was obtained in joint reading.

For the quantification of myocardial blood flow (MBF) in ml/g/min, left ventricular (LV) contours and input function region were acquired automatically with minimal operator intervention using Corridor4DM software (Cardiac; 4DM PET CFR) version 2016 (INVIA Medical Imaging Solutions, Ann Arbor, MI) [16, 20]. Initiating intravenous ^13^ N-ammonia injection, serial transaxial emission images were acquired (dynamic frames: 1 × 5 s, 12 × 10 s, 4 × 30 s, 4 × 60 s, 1 × 120 s) with PET/CT, and time-activity curves from the first 2 min in concert with a two-compartment tracer kinetic model were applied to quantify regional and global MBF in ml/g/min [2, 16]. On the polar map of the last 600-s image set, regions of interest (ROIs) were assigned to the territories of the main three coronary arteries using the 17-segment model. Using the Corridor4DM software package, MBF was quantified in ml/g/min. Regional MBFs of the LAD, LCx, and RCA territory were averaged on a polar map and the computed mean MBF of the left ventricle was defined as global MBF.

Subsequently, segmental MBF from the mid to mid-distal left ventricular myocardium was determined from the polar map to denote a MBF difference (ml/g/min) indicative of a longitudinal MBF gradient. Described in more detail, longitudinal MBFs in the mid and mid-distal myocardial segment of the LV corresponding to the vascular territories of the LAD (segments: 7–8 and 13–14), LCx (segments: 11–12 and 16), and RCA (segments: 9–10 and 15) were assessed [15]. Basal segments (LAD: 1–2, LCx: 5–6, and RCA: 3–4) and the apical segment (LAD: 17), however, were not considered for this evaluation due to possible count variability caused by the membranous septum, by a certain variability in locating the last apical slice, and by partial volume errors resulting from object size at the apex [15, 21]. Subsequently, regional longitudinal MBF gradients of the LAD, LCx, and RCA territory were then averaged on a polar map and the computed mean longitudinal MBF gradient of the left ventricle was defined as global longitudinal MBF gradient at rest and during hyperemic MBFs on a patient level. Further, alterations in the MBF difference from baseline to hyperemia were defined as Δ longitudinal MBF difference (MBF difference during hyperemia minus MBF difference at baseline) to signify Δ longitudinal MBF gradient [22]. In addition, applying receiver operating characteristic curve (ROC) for the identification of IEV by the PET-determined longitudinal MBF difference during stress flow stimulation yielded an optimal cutoff point of greater than 0.07 mL/g/min to distinguish between NEV and IEV with invasive measures as reference [23]. In view of the latter and prior PET flow studies (16,18), in the current study the absence of a longitudinal decrease in hyperemic MBF or mild decline within *<* −0.10 mL/g/min suggested the absence of a longitudinal MBF gradient (Fig. 1), whereas a value ≥ −0.10 mL/g/min was regarded as sufficient to denote an abnormal hyperemic longitudinal MBF gradient (Fig. 2).

Heart rate, blood pressure, and a 12-lead electrocardiogram were acquired continuously during each MBF measurement. From the mean heart rate and systolic blood pressure during the first 2 min of each image acquisition, the rate-pressure product (RPP) was derived as an index of cardiac work. Further, resting MBF was normalized to the RPP, and thus myocardial work (averaged during the first 2 min of image acquisition; MBF divided by RPP multiplied by 10 000). This again was applied to calculate the corrected MFR (Hyperemic MBF during regadenoson divided by NMBF at rest). From 8 bin -gated PET image acquisition, left ventricular end-diastolic volumes, end-systolic volumes, and resulting left ventricular ejection fraction (LVEF) were computed. In view of the low temporal resolution of 8-bin gated PET and systemic underestimation of the LVEF [24], a LVEF ≥ 45% was defined as normal given normal regional and global systolic function on visual analysis. In addition, assessment of intra-observer and inter-observer reproducibility was performed in ten randomly selected patients from group 1 with manifestation of hyperemic longitudinal MBF gradient. Data obtained were analyzed by reviewer 1 to determine the intra-observer reproducibility. A second observer, blinded to the findings of observer1, independently analyzed the image data, thus affording the assessment of the inter-observer reproducibility [25].

### Coronary artery calcifications estimate

Prior investigations [26] demonstrated a high agreement of visually estimated calcium artery score (VECAC) from low-dose and non-gated CT when compared to standard Agatston score (AS) from gated CT. A high degree of association was observed between VECAC and AS, with 63% of VECAC scores in exactly the same category as the AS category and 93% varying by no more than one category. Accordingly, we applied a visual four-point scoring system to denote coronary artery calcifications (0 = normal, 1 = mild, 2 = moderate, and 3 = severe calcifications) of the LAD, LCx, and RCA on the non-gated and low-dose CT for attenuation correction [2, 26]. From all three coronary vessels the mean coronary calcium score (CCS) was computed (2,22).

### Statistical analysis

Data are presented as the mean ± SD for quantitative and absolute frequencies for qualitative variables. For comparison of differences, appropriate t-tests for independent samples were used. A comparison of MBFs and MFR among the different groups was performed by 1-way analysis of variance (ANOVA) followed by Scheffe multiple comparison test. Differences in prevalence of gender, cardiovascular risk factors and coronary microvascular (dys)function among groups were analyzed with the x2 test. Multivariate analysis was performed by means of a linear stepwise regression model adjusting for the following a priori selected predictors of the hyperemic longitudinal MBF gradient: gender, CCS, global MFR, age, BMI, systolic blood pressure (SBP), total cholesterol, low-density lipoprotein (LDL) cholesterol, high-density lipoprotein (HDL) cholesterol, triglycerides, glucose, and high-sensitivity C-reactive protein (hsCRP). All test procedures were 2- tailed, and *p* ≤ 05 was considered statistically significant. All statistical analyses were performed with SPSS for Windows 27.0 (SPSS). For the assessment of intra- and inter-observer reproducibility (RPC) the standard deviation from the mean difference was multiplied by 1.96 to denote the range of differences between repeat measurements.

## Results

### Clinical characteristics

The clinical characteristics of the study population with predominant cardiometabolic risk constellation are denoted in Table 1. In the whole study population, 63% (210/332) had normal global MFR and thus coronary microvascular function, while 37% (122/332) presented abnormal reductions in global MFR to signify CMD. From these CMD, the classical or the endotype of CMF manifested in 25% (83/332) and 12% (39/332), respectively. As regards the prevalence of diffuse coronary artery calcification, it was 55% (181/332). When patients were subgroup, it was highest in G2 with 95% (79/83), followed by G3 with 49% (19/39) and G1 with 40% (83/210). Regarding the CCS, the extent of the CCS was significantly higher in G2 compared to G1 and non-significantly to G3 respectively, (*p* = 0.001 and *p* = 0.139, respectively). In addition, the extent of CCS was also higher in G2 than in G3 (*p* < 0.001). Thus, in the G2, the extent the CCS was highest among groups, while comparable between G1 and G3.

### Prevalence of IEV, hemodynamic findings, and MBF parameters

**Fig. 3:**
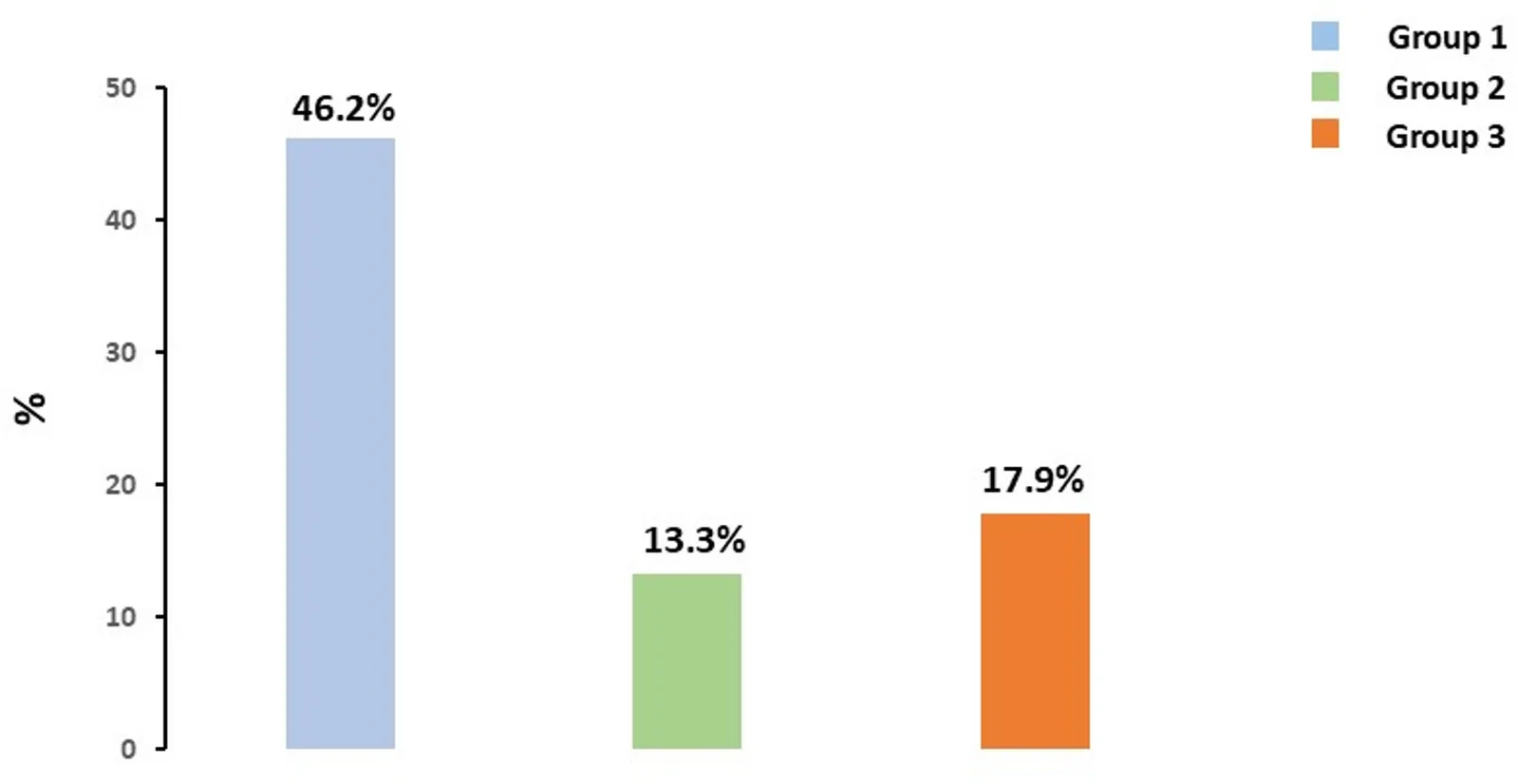
Prevalence and Distribution of Abnormal Hyperemic Longitudinal MBF Gradient among Groups. Highest prevalence of abnormal longitudinal MBF gradient or an impairment of flow-mediated epicardial vasodilation is observed in group 1 with nCMF, followed by group 2 with classical type of CMD and group 3 with endogen type of CMD, respectively

In the entire study population, IEV was present in 34.6.% (115/332). IEV was highest in group 1 with 46.2% (97/210), while there was a comparable low prevalence between group 2 and group 3 with 13.3% (11/83) vs. 17.9% (7/39) (*p* = 0.559) (Fig. 3). At rest, heart rate and systolic blood pressure and corresponding RPP did not differ significantly among G1 and G2, while they were the highest in G3 (Table 2). The latter was also accompanied by a progressive increase in global resting MBF and global normalized MBF from G1 to G2 and G3. During regadenoson-stimulated hyperemic flow increases, heart rates had increased significantly from rest, while SBP had decreased significantly. Conversely, stress SBP were highest in G3, and comparable between G1 and G2. This was widely mirrored by stress RPP among groups. Global hyperemic MBF was significantly higher in G1 than in G2, while comparable to G3, respectively. Conversely, global MFR was significantly higher in G1 when compared to G2 and G3, respectively, while it did not differ significantly between G2 and G3 (Table 2;  3). The group comparison of global hyperemic MBF did not reach statistical significance (*p* = 0.058 by ANOVA), while it was the case for global MFR among groups (*p* = 0.001 by ANOVA). When the global hyperemic MBFs were related to the mean arterial blood pressure to account for possible interindividual variations in coronary driving pressure, the resulting measures of global CVR (mean arterial blood pressure/global MBF) widely mirrored the global MBF values at rest and during pharmacologic vasodilation for G1-3 studied (Table 2). Consequently, differences in coronary driving pressure during pharmacologically stimulated hyperemic coronary flows can be widely excluded accounting for alterations in global hyperemic MBF responses. Also here, the group comparison of global hyperemic CVR among groups was significant (*p* = 0.001 by ANOVA).

**Table 2:** MBF and hemodynamic findings during PET/CT exam

Flow Parameters	Group 1	Group 2	*p* Value	Group 3	*p* Value
MBF-Rest	0.87 ± 0.18	0.90 ± 0.21	0.220	1.25 ± 0.13	0.001
NMBF-Rest	0.90 ± 0.20	0.92 ± 0.24	0.700	1.07 ± 0.16	0.001
MBF-Stress	2.39 ± 0.46	1.53 ± 0.30	0.001	2.32 ± 0.28	0.192
MFR	2.81 ± 0.51	1.73 ± 0.24	0.001	1.84 ± 0.13	0.001
Corrected MFR	2.73 ± 0.68	1.73 ± 0.41	0.001	2.21 ± 0.35	0.001
CVR-Rest	119 ± 25	117 ± 30	0.115	84 ± 10	0.001
CVR-Stress	37 ± 8	57 ± 13	0.001	41 ± 7	0.019
MBF Gradient-Rest	−0.03 ± 0.05	0.02 ± 0.09	0.113	−0.03 ± 0.03	0.578
MBF Gradient-Stress	−0.28 ± 0.10	−0.19 ± 0.08	0.008	−0.27 ± 0.10	0.795
∆MBF Gradient	−0.25 ± 0.09	−0.21 ± 0.09	0.028	−0.24 ± 0.09	0.234
CVR Gradient-Rest	3.43 ± 6.97	−1.83 ± 7.53	0.047	2.11 ± 1.64	0.172
CVR Gradient-Stress	3.77 ± 1.52	5.54 ± 1.99	0.001	3.89 ± 1.62	0.851
∆CVR Gradient	0.35 ± 6.49	7.37 ± 6.41	0.005	1.79 ± 1.96	0.561
Hemodynamics					
Rest HR, bpm	70 ± 11	70 ± 11	0.949	79 ± 9	0.001
Stress HR, bpm	92 ± 12	86 ± 13	0.001	96 ± 10	0.061
Rest-SBP, mmHg	139 ± 18	143 ± 23	0.128	151 ± 17	0.001
Stress-SBP, mmHg	121 ± 16	120 ± 22	0.881	133 ± 18	0.001
Rest-RPP	9793 ± 1974	10,056 ± 2122	0.330	12,014 ± 1810	0.001
Stress-RPP	11,218 ± 2137	10,364 ± 2806	0.005	12,783 ± 2136	0.001
∆RPP, pharmacologic – rest	1425 ± 1452	307 ± 1365	0.001	768 ± 1422	0.010

Values are mean ± standard deviation (SD). P values versus Group1 (Mann-Whitney U test for independent samples)

CVR = coronary vascular resistance; HR = heart rate; MBF = myocardial blood flow; MFR = myocardial flow reserve

PET = positron emission tomography; RPP = rate-pressure product; SBP = systolic blood pressure

### Longitudinal MBF gradient

**Fig. 4:**
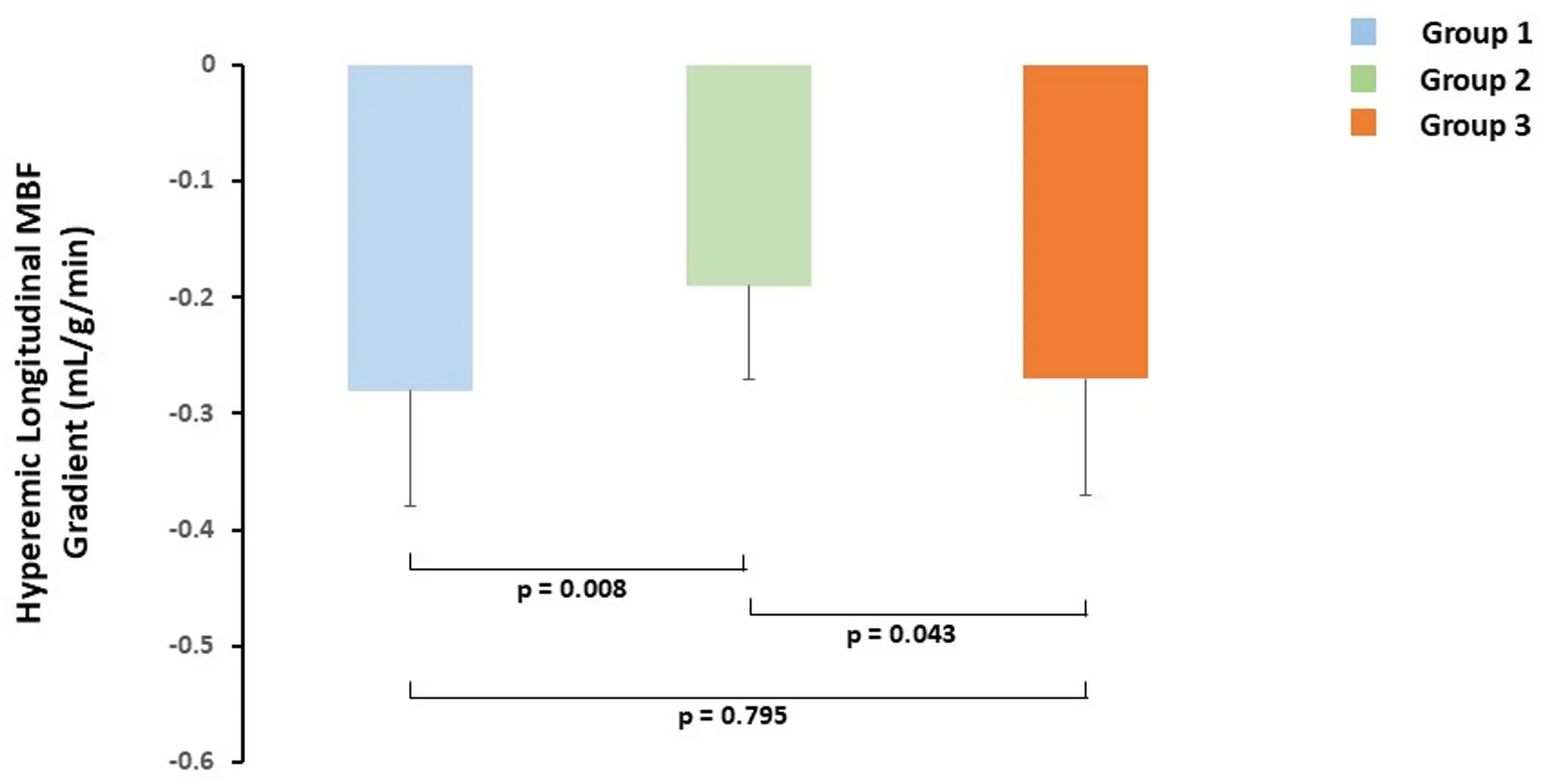
Hyperemic Longitudinal MBF Gradient among Groups. As can be appreciated, the extent of abnormal hyperemic longitudinal MBF gradient was more pronounced in group 1 with nCMF and group 3 with endogen type of CMD when compared to group 2 with classical CMD, while it was comparable between group 1 and group 3

There was virtually no longitudinal MBF gradient at rest among groups (Table 2). During pharmacologically induced hyperemia, the observed hyperemic longitudinal MBF gradient was significantly higher in G1 and G3 when compared to G2, respectively, whereas it was comparable between G1 and G3 (*p* = 0.795) (Fig. 4). Similarly, the change in longitudinal MBF gradient from rest to hyperemia defined as Δ longitudinal MBF gradient (longitudinal MBF gradient during hyperemia minus longitudinal MBF gradient at rest) was significantly higher in G1 and G3 when compared to G2 in comparison to G2, respectively, while it did not differ significantly between comparable between G1 and G3 (*p* = 0.111) (Table 2). The group comparison of the hyperemic longitudinal MBF gradient as well as of the Δ longitudinal MBF gradient differences was significant among groups, respectively (*p* = 0.029 and *p* = 0.008 by ANOVA, respectively).

**Figs. 5:**
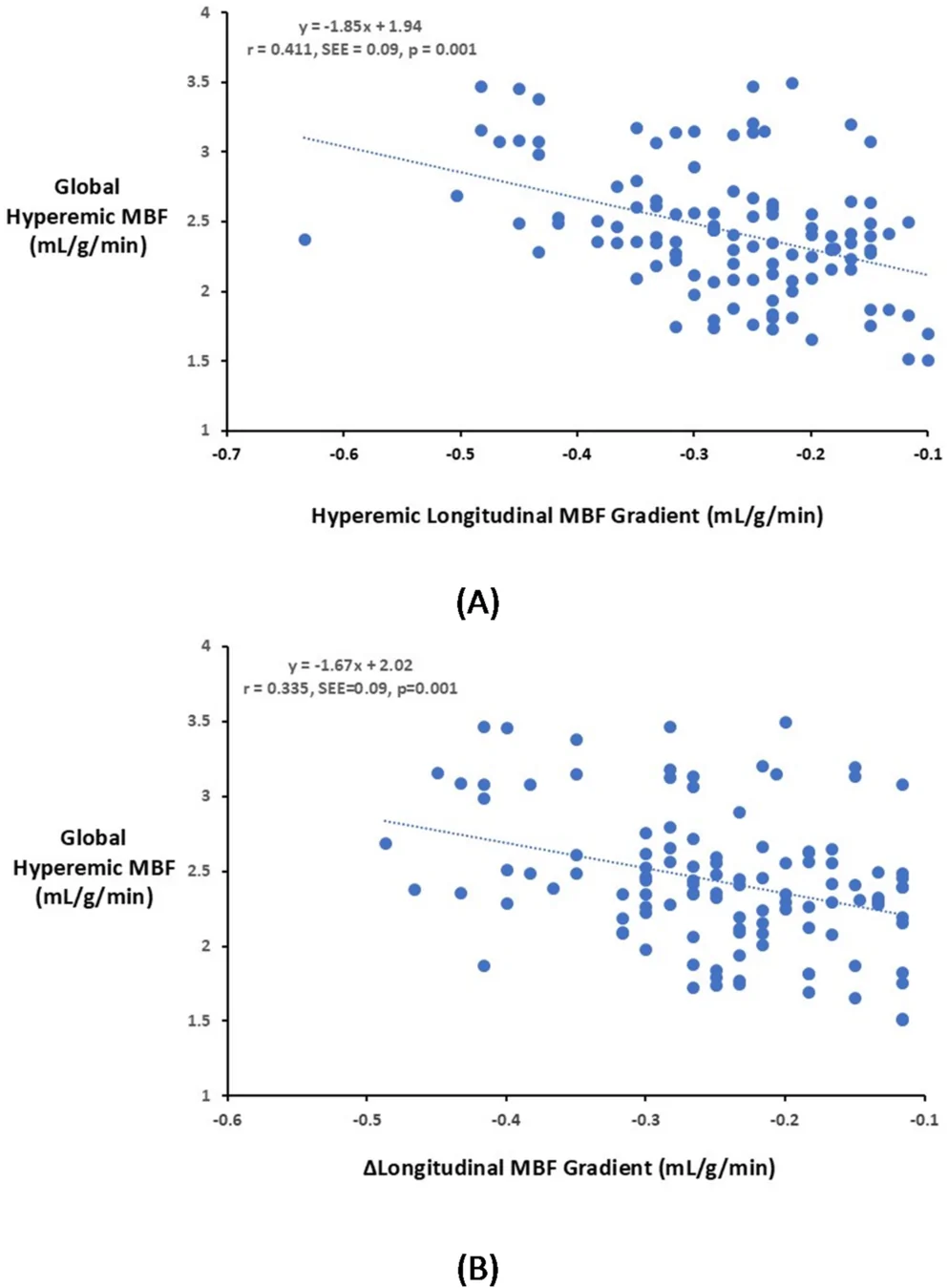
Correlation between Global Hyperemic MBF and MBF Gradients. Figure 5a denotes the correlation between global hyperemic MBF and corresponding abnormal longitudinal MBF gradient. In addition, Fig. 5b demonstrates that the global hyperemic MBF also associates with the corresponding ∆longitudinal MBF gradient (longitudinal MBF gradient during pharmacologic stress - longitudinal MBF gradient at rest)

**Figs. 6:**
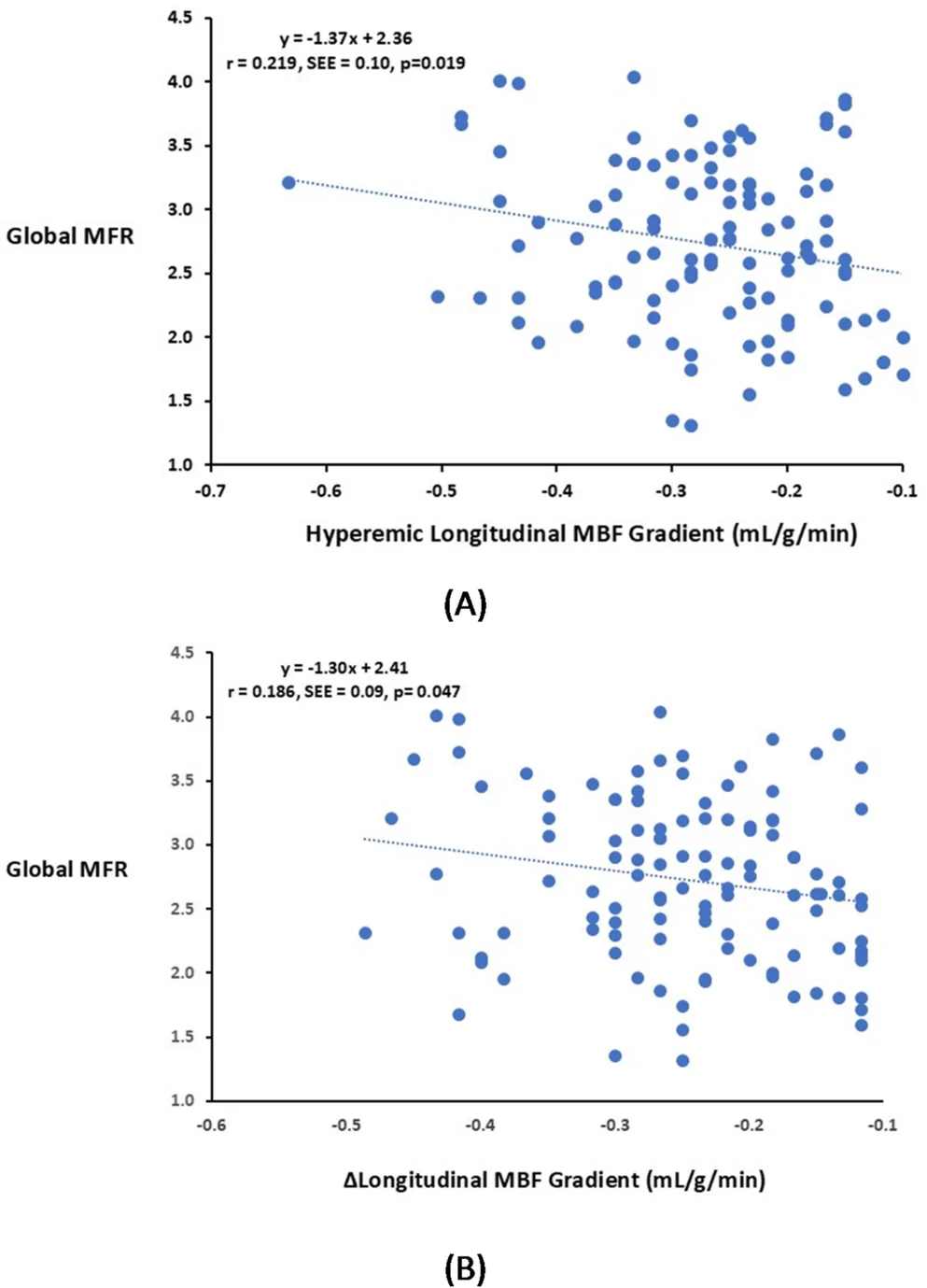
Correlation between Global Myocardial Flow Reserve (MFR) and MBF Gradients. Figure 6a unravels a significant association between the global MFR and corresponding abnormal hyperemic longitudinal MBF gradient. Further, Fig. 6b signifies that the global MFR also correlates with the corresponding ∆longitudinal MBF gradient (longitudinal MBF gradient during pharmacologic stress - longitudinal MBF gradient at rest). Central Illustration: Assessment of Global MBF and Hyperemic Longitudinal MBF Gradient with ^13^N-ammonia PET/CT Legend: nCMF = normal coronary microvascular function; CMD = coronary microvascular dysfunction, group 1 = normal coronary microvascular function; group 2 = classical type of CMD; group 3 = endogen type of CMD; MFR = myocardial flow reserve; MBF = myocardial blood flow

To evaluate a possible relationship between the longitudinal MBF gradient and coronary microvascular function, the hyperemic MBF gradient Δ longitudinal MBF gradient was compared to the corresponding global hyperemic MBF and global MFR, respectively. As can be appreciated from Figs. 5a-b, the global hyperemic MBF correlated inversely with the hyperemic longitudinal MBF gradient and the Δ longitudinal MBF gradient, respectively, (*r* = 0.411, SEE = 0.09, *p* = 0.001 and *r* = 0.335, SEE = 0.09, *p* = 0.001). Similarly, there was a significant and inverse association between global MFR and the hyperemic longitudinal MBF gradient and the Δ longitudinal MBF gradient, respectively (*r* = 0.219, SEE = 0.10, *p* = 0.019 and *r* = 0.186, SEE = 0.09, *p* = 0.047) (Figs. 6a-b). These observations denote an increase in coronary flow as a determinant of the longitudinal MBF gradient during hyperemic flows. Finally, on univariate analysis, only global MFR and age were significantly associated with the hyperemic longitudinal MBF gradient (Table 3). Conversely, on multivariate analysis, only BMI proved to be an independent predictor for the hyperemic longitudinal MBF gradient.

**Table 3:** Predictors of hyperemic abnormal MBF gradient

	Univariate	Multivariate Analysis	
	*p* Value	Standardized Coefficient	*p* Value
CCS	0.160	/	/
Global MFR	**0.019**	/	/
Gender	0.064	/	/
BMI, kg/m2	0.113	**0.440**	**0.004**
Age, yrs	**0.048**	/	/
SBP at rest, mmHg	0.122	/	/
Total cholesterol, mg/dl	0.707	/	/
LDL cholesterol, mg/dl	0.891	/	/
HDL cholesterol, mg/dl	0.770	/	/
Triglyceride, mg/dl	0.248	/	/
Glucose, mg/dl	0.380	/	/
Hs CRP, mg/l	0.098	/	/
HbA_1c_, %	0.439	/	/

* Significant difference by analysis of variance

CCS = coronary artery calcium score; MFR = myocardial flow reserve; BMI = body mass index;

HbA1c = glycosylated hemoglobin; HDL = high-density lipoprotein; hsCRP = high-sensitive

C-reactive; protein; LDL = low-density lipoprotein; SBP = systolic blood pressure

### Reproducibility of longitudinal MBF

In 10 randomly selected patients of group 1 with hyperemic longitudinal MBF gradient manifestation the mean global hyperemic MBF increased to 2.45 ± 0.48 from 0.81 ± 0.20 mL/g/min at rest resulting in a global MFR of 3.09 ± 0.42. Further, mean values for repeat evaluation of the longitudinal MBF gradient the by one observer were nearly identical at rest with a mean difference of 0.007 mL/g/min (Table 4). During pharmacologic stress, mean values were widely comparable to rest with a mean difference of 0.007 mL/g/min, while the mean difference for ∆longitudinal MBF gradient was 0.017 mL/g/min. This resulted in intra-observer repeatability coefficient (RPC) of 3.5% at rest, 2.7% during pharmacologic stress, and for the ∆longitudinal MBF gradient 7.5% respectively. When available data were analyzed by two different observers the mean difference of MBF values for the longitudinal MBF gradient at rest, during pharmacologic, and ∆change (stress-rest) were 0.008, 0.015, and 0.012, respectively. This resulted in corresponding RPC of 5.6%, 5.1% and 3.9% respectively.

**Table 4:** Repeat longitudinal MBF gradient at measurements and mean differences

At rest	M1	M2	M3	Mean difference 1–2	Mean difference 1–3
	−0.023 ± 0.040	−0.023 ± 0.043	−0.018 ± 0.043	0.007 ± 0.009	0.008 ± 0.014
During stress	−0.258 ± 0.088	−0.252 ± 0.083	−0.263 ± 0.099	0.007 ± 0.021	0.015 ± 0.015
∆ Change (stress- rest)	−0.235 ± 0.088	−0.228 ± 0.092	−0.245 ± 0.093	0.017 ± 0.014	0.012 ± 0.018

Values are mean ± standard deviation (SD). Measurements 1 = M1; measurements 2 = M2; measurements 3 = M3; MBF = myocardial blood flow; PET = positron emission tomography

### Discussion

The results presented in the current study are unique as it unravels that nearly half of symptomatic patients with normal rest-stress myocardial perfusion imaging and normal global MFR had impaired flow-mediated and thus endothelial dysfunction of the epicardial artery in a study population with a predominant cardiometabolic risk profile.

Notably, the hyperemic MBF gradient was significantly and inversely associated with the global hyperemic MBF, denoting indeed increases in coronary blood flow as a pivotal determinant of the longitudinal MBF gradient during hyperemic flows.

It is important to distinguish between specific identification of endothelium-dependent and – independent epicardial and coronary microvascular function during invasive coronary angiography with e.g. acetylcholine-stimulated endothelial release of nitric oxide and a non-invasive probe of a flow-dependent hemodynamic phenomenon with PET-determined abnormal hyperemic longitudinal MBF likely related to an impairment of flow-mediated epicardial vasodilation in the presence of diffuse CAD and/or early vasomotor dysfunction of the epicardial artery [14, 27]. The observed abnormal hyperemic longitudinal MBF gradient, however, accords with prior studies for obstructive and nonobstructive CAD in individuals with cardiovascular risk factors [15, 22, 28, 29]. Such abnormal decrease in hyperemic longitudinal MBFs has been connected to downstream fluid dynamic effects of CAD-induced diffuse luminal narrowing and/or functional alterations of the epicardial coronary conduit vessels [2, 14, 21, 30, 31]. Notably, increases in global hyperemic MBFs and global MFR were accompanied by a progressive increase in the magnitude of the longitudinal MBF gradient in the presence of predominantly non-obstructive CAD. These observations may indeed accord with the Hagen–Poiseuille and described in previous studies [15, 21, 30]. According to this, the resistance to hyperemic flows is stringent on the vessel length, the flow velocity and, inversely on the fourth power of the arterial diameter. This concept accords with the current findings in patients without focal and hemodynamically obstructive CAD lesions, signifying a coupling of the extent of the longitudinal MBF gradient to alterations in MFR in the presence of diffuse CAD and/or functional alterations of the epicardial coronary conduit vessels as also reported previously [2, 14, 31].

Yet, it is important to realize that in the presence of the classical type of more severe CMD in the current study population, associated with markedly reduced global hyperemic MBFs, the magnitude of the longitudinal MBF gradient may have been widely offset, leading to a less pronounced or absence of a gradient even in the presence of moderate severe epicardial artery calcifications. This maybe reflected also the reported low prevalence of IEV of only 13% in patients with classical CMD. Thus, the lower prevalence of IEV in the classical CMD group may simply reflect the fact that these patients have had reduced hyperemic flow and therefore generate a less pronounced longitudinal gradient, rather than indicating relatively preserved epicardial function. Conversely, the higher prevalence of IEV in the group with normal MFR may partly reflect preserved hyperemic flow conditions that make the gradient easier to detect. Thus, in the presence of the classical type of CMD, IEV may ineed be missed with with PET flow quantification and the study may be seen as incomplete. These observations may stimulate further invasive and comparative studies with the specific assessment of endothelium-dependent epicardial vasomotor function with for example acetylcholine testing to evaluate the diagnostic gap of PET-determined IEV in patients presenting the classical type of CMD. Whether the diagnostic gap of PET-determined IEV in the presence of classical type of CMD leads to a suboptimal prognostication in these patients neccesitates further exploration.

Interestingly, the prevalence of IEV in the presence of endogen type of CMD was also quite low with 18% despite the presence of high hyperemic MBFs. At first sight this may be surprising, but it may be related to a shift in a less atherogenic lipid profile, inflammatory-metabolic environment, alterations in adipocytokine pattern, and/or increases in circulating progenitor with vascular repair to marked elevations in subcutaneous adipose tissue leading to less vulnerability of the epicardial artery maintaining normal flow-mediated and, thus, endothelium-dependent epicardial vasomotor function in patients with severe obesity [16, 20]. Another important consideration is that most of these patients with the endogen type of CMD had hypertensive arterial blood pressure regulation in the presence or absence of advanced obesity leading to increases in resting MBFs as predominant cause of abnormal reductions in global MFR in the absence of IEV. In addition, the duration of exposure to cardiometabolic risk may not have been sufficiently long enough to initiate and advance diffuse CAD and/or functional alterations of the epicardial coronary conduit vessels in these patients with the endogen type of CMD. Such sustained and endothelium-dependent epicardial vasomotor function may also translate into less CAD plaque burden in these study patients with endogen type of CMD than in those with the classical type of CMD or even normal global MFR, respectively, as reflected by current results of CT-determined coronary artery calcifications.

Pharmacologically induced hyperemia with regadenoson induces hyperemic MBF increases through vascular smooth muscle cell relaxation of the arteriolar vessels. In this respect, the resulting global hyperemic MBF increase is seen as a non-invasive probe of predominant endothelium-independent flow response or coronary circulatory function. Of note, this increase in intracoronary flow stimulates shear- sensitive components of the coronary endothelium contribute by about 20% to 25% through flow-mediated coronary vasodilation to the global hyperemic MBF increases [32, 33]. Epicardial vessel stiffening through diffuse CAD and/or functional impairment of the coronary endothelium, however, may in fact prevent the flow-mediated epicardial vasodilation during higher flows leading to down-stream fluid dynamic hampering effects that may manifest as an abnormal longitudinal hyperemic MBF decrease from the base-to-apex direction in cardiovascular risk individuals. Given such constraints, the resistance to higher coronary flow paradoxically increases resulting in a progressive proximal-to-distal decrease in intracoronary pressure along the epicardial artery [34], or into an abnormal longitudinal MBF gradient as described and validated in prior PET flow studies [2, 14–16, 21]. As observed in the current and previous investigation [2], the severity of IEV, reflected by the extent of the hyperemic longitudinal MBF gradient, was significantly more pronounced in patients with normal hyperemic flow increases than in those with predominant classical CMD and thus reduced global hyperemic flow increases. This observation in fact accords with the Hagen-Poiseuille law [14, 15, 22], that denotes the resistance to flow dependent on the length of the epicardial artery, the flow velocity and, notably, inversely on the fourth power of the vessel diameter. In the current entire study population, the extent of the hyperemic longitudinal MBF gradient closely and inversely associated with the degree of global hyperemic MBF increases, so that higher coronary flow increases were indeed paralleled with a more severe hyperemic longitudinal MBF gradient, signifying downstream effects of IEV on flow owing to diffuse CAD and/or functional alterations of the epicardial artery.

Interestingly, advanced age likely associated with a longer period of exposure to cardiovascular risk factors clustered by the metabolic syndrome can be assumed to have contributed to the observed hyperemic abnormal longitudinal MBF gradient. By multivariate analysis, however, only BMI proved to be independent predictors of the hyperemic longitudinal MBF gradient. The independent predictive value of BMI for the hyperemic longitudinal MBF gradient may put forth complex interactions among components of the metabolic syndrome clustering adverse effects of arterial blood pressure, insulin resistance, and microinflammation on the coronary endothelium favoring an impairment of flow-mediated epicardial vasodilation. As invasive studies of epicardial vasomotor function [3, 4] have demonstrated that the identification of IEV confers an increased risk in future cardiovascular events, current observations may indeed suggest PET-determined IEV as a non-invasive mechanistic link between coronary circulatory function and cardiovascular outcomes, deserving further clinical outcome testing. If such considerations hold true, then the longitudinal MBF gradient may serve as a novel indicator for coronary flow regulation and cardiovascular prognostic assessment.

Interestingly, the prevalence of non-gated CT-determined coronary artery calcifications, as a reflection of coronary artery plaque burden, in the entire study population was 55%. Thus, despite a widely comparable cardiometabolic risk profile among groups, diffuse CAD was present in about half of these patients. Interestingly, when patients were subgrouped, the presence of coronary artery calcifications (CAC) was highest in the group classical type of CMD with 95%, followed by comparable manifestation of CAC in patients with normal coronary microvascular function and those with the endogen type of CMD (40% and 49%, respectively). When looking at the severity of CAC, it was moderately severe in the classical type of CMD, while mild and comparable between patients with normal coronary microvascular function and the endogen type of CMD. Thus, it is intriguing to speculate that in the current study, the presence of more moderate severe diffuse CAD in the classical type of CMD may also reflect a more advanced stage of function alterations of the coronary microvascular function, needing furthermore large-scale investigations. These observations, however, may outline that apart from IEV as functional substrate of epicardial vessel stiffness, early structural alterations or diffuse CAD may also be associated with the described hyperemic longitudinal MBF gradient in this cardiometabolic cardiovascular risk population and as also reported in previous clinical investigations [2, 14, 15, 30].

### Limitation

There are several important limitations to be considered in interpretation of the current study. First, the study analysis was conducted in a cardiometabolic risk population. Thus, further clinical investigations of population with different cardiovascular risk profiles are needed. Second, we only performed a non-gated CT for attenuation correction of PET emission data instead of a dedicated and gated CT for a more sensitive and accurate identification and quantification of the coronary artery calcium score. Thus, some mild coronary artery calcifications may not have been detected that have likely resulted in a systemic and relative underestimation of reported coronary calcification. Third, since no contrast CT coronary angiography or invasive intravascular coronary ultrasound was conducted, early and subclinical CAD likely may have been missed, that may have accounted for the reported IEV in those individuals without coronary artery calcifications. Fourth, the abnormal hyperemic longitudinal MBF gradient was quantified from the mid and mid-to-distal left ventricular segments along a relatively short longitudinal distance striving to avoid confounding count variability in the basal segments and partial volume effects in the apical segment on flow measurements with current MBF quantification software [2]. Consequently, the measured hyperemic longitudinal MBF gradient in the current study therefore is likely underestimated as reported by others [14, 21]. Fifth, intra- and interobserver reproducibility PET flow studies for the assessment of the measurement error of the hyperemic longitudinal MBF gradient in healthy controls and cardiovascular risk individuals are still needed. Sixth, given the relatively small range of alterations in segmental or territorial hyperemic longitudinal MBFs, we evaluated the averaged MBF difference or gradient across the territories of the LAD, LCx, and RCA distribution on a patient level to provide more consistent and reproducible values of these segmental MBF measurements. In this respect, novel approached with mapping MBFs on a pixel basis allowing someone to measure the longitudinal flow gradient over a longer distance from the basal to apical segments likely affording also a more reliable analysis of territorial longitudinal MBF gradients and rendering it clinically more applicable, needing further investigations [17, 27]. Finally, further comparative invasive angiographic and PET flow studies are still needed to identify the optimal threshold of a hyperemic longitudinal decrease in MBFs for the identification of IEV and characterization of its severity.

## Conclusions

Nearly half of these patients with normal global MFR may have abnormal hyperemic longitudinal MBF gradient likely to reflect an impairment of flow-mediated epicardial vasodilation in a study population with a predominant cardiometabolic risk profile. In the presence of normal coronary microvascular function, the reported hyperemic longitudinal MBF gradient is likely to add a new and pivotal flow parameter in the assessment of coronary flow modulation and cardiovascular prognostication warranting further clinical testing.

## Data Availability

No datasets were generated or analysed during the current study.

## Abbreviations

CAD: coronary artery disease
CMD: coronary microvascular dysfunction
CT: computed tomography
CVR: coronary vascular resistance
HDL: high-density lipoprotein
LDL: low-density lipoprotein
hsCRP: high-sensitivity C-reactive protein
LV: left ventricular
MBF: myocardial blood flow
MFR: myocardial flow reserve
PET: positron emission tomography
RPP: rate-pressure product
